# Effects of comorbid alexithymia on cognitive impairment in chronic schizophrenia: a large-sample study on the Han Chinese population

**DOI:** 10.3389/fpsyt.2025.1517540

**Published:** 2025-03-06

**Authors:** Yuanping Liao, Yunhui Zhong, Kan Yang, Xiang-Yang Zhang

**Affiliations:** ^1^ The Third People’s Hospital of Ganzhou, Ganzhou, China; ^2^ Department of Psychology, Gannan Medical University, Ganzhou, China; ^3^ CAS Key Laboratory of Mental Health, Institute of Psychology, Chinese Academy of Sciences, Beijing, China; ^4^ Department of Psychology, University of Chinese Academy of Sciences, Beijing, China

**Keywords:** schizophrenia, alexithymia, cognitive impairment, RBANS, comorbid

## Abstract

**Background:**

Alexithymia and cognitive dysfunction are common in patients with schizophrenia. However, only a few studies have investigated the cognitive performance of patients with schizophrenia and comorbid alexithymia. This study aimed to investigate the relationship between alexithymia and neurocognitive impairment in patients with schizophrenia.

**Methods:**

A total of 695 patients who met the DSM-IV diagnostic criteria for schizophrenia were included in this cross-sectional study (male/female = 464/231). Demographic and clinical data were collected using self-reported questionnaires. The severity of alexithymia was assessed using the Toronto Alexithymia Scale (TAS-20), cognitive function was assessed using the Repeatable Battery for the Assessment of Neuropsychological Status (RBANS) tool, and the severity of psychiatric symptoms was assessed using the Positive and Negative Syndrome Scale (PANSS).

**Results:**

The prevalence of comorbid alexithymia in patients with chronic schizophrenia was 31.40%, with a male preponderance. Patients with alexithymia had higher PANSS negative symptom subscale scores and PANSS total scores than those without alexithymia (*p* < 0.05 for all). In addition, patients with alexithymia had more severe deficits in immediate memory, delayed memory, and language and lower RBANS scores than those without alexithymia. Stepwise multivariate regression analysis showed that alexithymia was a risk factor for language deficits and indicated low total RBANS scores in patients with schizophrenia.

**Conclusion:**

This study suggests that patients with chronic schizophrenia with alexithymia have poorer cognitive function than those without alexithymia. Some demographic characteristics and alexithymia are risk factors for cognitive dysfunction in patients with chronic schizophrenia.

## Introduction

Alexithymia was initially introduced by Sifneos in 1973 within the context of psychosomatic disorders (1). It is clinically characterized by the inability to verbally articulate emotions, an absence of inner fantasy, the use of mundane and specific words and thoughts predominantly associated with external events, a tendency to describe physical symptoms over emotions, difficulty recognizing internal emotions or bodily sensations, and the inability to use these sensations or feelings as indicators of emotional distress (2). Studies have highlighted a strong relationship between alexithymia and schizophrenia (SCZ) (3). Compared with healthy individuals, patients with SCZ have a higher risk of developing alexithymia (4). Yi et al. reported that the prevalence of alexithymia in patients with SCZ was as high as 35.2% (5). Notably, alexithymia has a strong influence on psychopathological conditions (6), sleep quality (7), suicidal ideation (8), social functioning (4), and neurocognition (9) in patients with SCZ.

Neurocognition, defined as the process of connecting and evaluating information, encompasses processing speed, attention, verbal and visual learning, memory, working memory, and reasoning and problem-solving skills (10). Approximately 75% of patients with SCZ experience cognitive impairment (11), which is not only a core feature of the disorder (12–14) but also a strong predictor of functional outcomes (15). This impairment can lead to compromised work and social functioning, resulting in unemployment and an inability to live independently (16, 17).

Some studies have shown a relationship between alexithymia and neurocognitive impairment, particularly in memory and executive functioning (18–20). Hyper-referential alexithymia has been identified as a risk factor for cognitive decline (12). However, existing studies on the relationship between alexithymia and neurocognition in patients with SCZ have shown inconsistent findings. For instance, Fogley et al. discovered that alexithymia, specifically difficulty identifying feelings (DIF) and externally oriented thinking (EOT) rather than difficulty describing feelings (DDF), was associated with processing speed and working memory in patients with SCZ (18). On the contrary, He et al. found that DIF, DDF, and EOT in alexithymia were correlated with poorer visuospatial functioning, executive functioning, and delayed memory but not with verbal functioning (21). Moreover, Peng et al. demonstrated an independent relationship between language and DDF (9). These divergent findings emphasize the requirement for a comprehensive investigation into the relationship between alexithymia and neurocognition.

To address these inconsistencies, the current study aims to more comprehensively examine the relationship between alexithymia and neurocognitive performance in patients with chronic SCZ. By including a large sample of 695 patients and utilizing validated measures such as the Toronto Alexithymia Scale (TAS-20) for alexithymia, the Repeatable Battery for the Assessment of Neuropsychological Status (RBANS) for cognitive function, and the Positive and Negative Syndrome Scale (PANSS) for symptom severity, this study provides a more robust analysis of the potential impact of alexithymia on various cognitive domains, including memory, attention, and language.

In this study, we hypothesize that patients with SCZ and comorbid alexithymia will exhibit more severe cognitive impairment, particularly in memory and language, than those without alexithymia. By exploring this hypothesis, we aim to provide a clearer understanding of how alexithymia contributes to cognitive deficits in chronic schizophrenia, thereby informing both clinical interventions and future research in this area.

## Methods

### Study design and participants

This cross-sectional study recruited 720 patients with chronic schizophrenia (SCZ) from Wuhan Xinzhou Mental Health Center and Guangzhou Huiai Hospital, China, from January to April 2019. The data used in this study were shared with a previously published paper by the last author (DOI: 10.30773/pi.2023.0004), which also utilized data collected under the same ethical approval from the Institutional Review Board of the Institute of Psychology, Chinese Academy of Sciences (IRB: H18031). The cohort used in both studies includes patients with chronic SCZ diagnosed based on the Chinese version of the Structured Clinical Interview for Diagnostic and Statistical Mental Disorders-5 (DSM-5), and all participants had signed informed consent forms.

The data collection protocols were consistent with those described in the previously published paper (DOI: 10.30773/pi.2023.0004), and all participants or their legal guardians provided written informed consent prior to participation.

### Clinical interview and measurements

#### Sociodemographic characteristics

A data collection form was used to obtain information regarding the demographic and clinical characteristics of the participants, including age, sex, level of education, marital status, BMI, age at the onset of SCZ, duration of the illness, and smoking behavior. BMI was calculated using the formula: BMI = weight (kg)/height (migh Height and weight were measured by trained researchers using standardized procedures. A questionnaire was administered by a researcher through a one-on-one semi-structured interview. Subsequently, the researcher collected the clinical data of patients by reviewing their medical records and conducting clinical interviews with them.

#### Assessment of the severity of alexithymia

The Toronto Alexithymia Scale (TAS-20) was used to assess the severity of alexithymia (22). TAS-20 is a self-reporting tool widely used to evaluate the severity of alexithymia in various populations, including patients with SCZ (18, 23). The responses are scored on a scale of 1–5 (from “strongly disagree” to “strongly agree”, respectively). The total score on the scale ranges from 20 to 100, with higher scores indicating higher severity of alexithymia. In this study, patients with a TAS-20 score of >60 were considered to have alexithymia. TAS-20 has demonstrated good internal consistency and retest reliability in previous studies (22). In this study, the Cronbach’s α value for total TAS-20 scores was 0.86.

#### Clinical assessment

The severity of clinical psychiatric symptoms was assessed using the Positive and Negative Syndrome Scale (PANSS) (24). PANSS traditionally contains three subscales, namely, positive symptom, negative symptom, and general psychopathology. To ensure the reliability and consistency of the scores, two psychiatrists from our team attended a training program on the use of PANSS. In repeated assessments, the inter-observer correlation coefficient for the total PANSS score was maintained at >0.8. Higher scores indicated more severe symptoms. PANSS has been demonstrated to have strong construct validity and has been widely used in patients with SCZ (25).

#### Cognitive assessment

Cognitive function was assessed using the Repeatable Battery for the Assessment of Neuropsychological Status (RBANS, Form A). The RBANS tool consists of 12 subtests used to calculate five age-adjusted index scores and a total score. The five test indices include immediate memory, visuospatial/constructional skills, language, attention, and delayed memory. We provided appropriate training to each patient so that they could adapt to the testing environment and the computerized task. In repeated assessments, the intra-rater correlation coefficient was 0.92.

### Statistical analysis

Continuous demographic and clinical variables were compared between patients with and without alexithymia using analysis of variance (ANOVA), whereas categorical variables were compared using the chi-square test. In addition, The correlation coefficient matrix between Cognitive performance and alexithymia was calculated using Pearson correlation analyses on each RBANS score and the matching TAS-20 total scores and subscales. The Bonferroni method was used to adjust for multiple tests. We then performed partial correlations by controlling covariates (including sex, Negative symptom and PANSS Total score) and investigating the interconnections between Cognitive performance and alexithymia. Binary logistic regression analysis was performed to identify risk factors closely associated with comorbid alexithymia. Furthermore, stepwise multiple regression analysis was used to examine the relationship between the total TAS-20 score and the PANSS or RBANS score.

All data were statistically analyzed using the SPSS (version 21.0) software (IBM, Armonk, NY, USA) and were expressed as the mean ± standard deviation (mean ± SD). A two-tailed p-value of <0.05 indicated statistically significant differences.

## Results

### Demographic and clinical characteristics of the participants

A total of 25 patients who refused to participate in the study or whose scales were filled incompletely were excluded from this study. Finally, a total of 695 patients with SCZ, including 464 men and 231 women, were included in this study. The duration of the illness of the included patients was 20.69 ± 11.80 years. The mean age of the included patients was 45.95 ± 12.41 years, ranging from 20 to 70 years. The mean duration of education was 9.49 ± 3.17 years, ranging from 1 to 21 years. The mean age at the onset of SCZ was 25.26 ± 7.56 years, ranging from 9 to 63 years. The mean BMI was 24.797 ± 4.44, ranging from 14.88 to 56.24. On the PANSS, the mean scores for positive symptom, negative symptom, and general psychopathology were 15.90 ± 5.23, 20.49 ± 6.39, and 38.82 ± 8.14, respectively, whereas the mean total score was 75.21 ± 16.30.

### Prevalence of alexithymia in patients with SCZ and the correlation between the two conditions

As shown in **Table 1**, 218 (31.40%) of the 695 patients with SCZ had comorbid alexithymia. A significant sex difference was observed in the prevalence of alexithymia, with the proportion of male patients being higher than that of female patients (35.34% versus 23.37%; χ^2^ = 10.261, p = 0.001). After age and educational qualification were controlled for, the prevalence rate of alexithymia was 0.56 times higher in men than in women (B = 0.583, Wald statistic = 10.125, p < 0.001, OR = 0.558, 95% CI = 0.390–0.799). However, no significant differences in any other demographic variables were observed between patients with and without alexithymia (p > 0.05 for all). Compared with patients without alexithymia, those with alexithymia had higher PANSS negative symptom scores (p < 0.001) and total PANSS scores (p = 0.012). Furthermore, a logistic regression model showed that sex (Wald χ^2^ = 9.496, df = 1, p < 0.01) and PANSS negative symptom scores (Wald χ^2^ = 20.807, df = 1, p < 0.01) were independent factors contributing to comorbid alexithymia. Correlation analysis indicated that the total TAS-20 score was significantly positively associated with the PANSS negative symptom score (r = 0.24, p < 0.001), PANSS general psychopathology score (r = 0.14, p < 0.001), and total PANSS score (r = 0.17, p < 0.001). These correlations remained significant after Bonferroni correction (p < 0.001 for all). Subsequently, multiple regression analysis validated that the total TAS-20 score was significantly associated with the PANSS negative symptom score (β = 0.26, t = 2.91, p < 0.01).

**Table 1:** Characteristics of schizophrenia patients with or without Alexithymia.

Characteristics	Total patients	With Alexithymia	Without Alexithymia	*F*/χ^2^	*df*	*P*-value
	695	(n=218, 31.40%)	(n=477, 68.60%)			
Age (years)	45.95 (± 12.41)	46.20 (± 12.06)	45.83 (± 12.57)	0.13	1,69	0.714
Education (years)	9.49 (± 3.17)	8.695 (± 1.63)	8.344 (± 1.17)	2.94	1,693	0.087
Gender (Male/Female)	464/231	164/54	300/177	10.26	1	**0.001**
Marital status				0.47	3	0.927
Single	429	135	294			
Married	148	46	102			
Divorced	111	34	77			
duration of the illness	20.69(± 11.80)	20.85 (± 12.07)	20.61 (± 11.69)	0.062	1	0.803
Previous hospitalizations	3.53 (± 2.31)	3.81 (± 2.46)	3.41 (± 2.22)	4.541	1	0.033
Losing spouse	7	3	4			
Never Smoker/Former Smoker/Current Smoker	100/103/192	115/42/61	285/61/131	4.75	2	0.093
Age at onset (years)	25.26 (± 7.56)	25.35 (± 7.87)	25.22 (± 7.42)	0.05	1,693	0.833
Age of hospitalization at onset (years)	28.20 (± 9.71)	29.09 (± 10.41)	27.79 (± 9.36)	2.66	1,693	0.103
BMI	24.797 (± 4.44)	24.52 (± 4.035)	24.92 (± 4.61)	1.21	1,693	0.272
Family history						
Yes	118	32	86	1.19	1	0.275
No	577	186	391			
PANSS						
Positive symptom	15.90 (± 5.23)	15.55 (± 4.68)	16.06 (± 5.47)	1.44	1,693	0.231
Negative symptom	20.49 (± 6.39)	22.29 (± 6.90)	19.67 (± 5.96)	26.23	1,693	**0.000**
General psychopathology	38.82 (± 8.14)	39.66 (± 8.41)	38.43 (± 7.99)	3.41	1,693	0.065
Total score	75.21 (± 16.30)	77.50 (± 16.80)	74.16 (± 15.98)	6.34	1,693	**0.012**

Significant differences are highlighted in bold.

BMI, body mass index; PANSS, the Positive and Negative Syndrome Scale.

### Cognitive performance of patients with and without alexithymia

**Table 2** shows the cognitive performance of patients with and without alexithymia evaluated using ANOVA. Compared with patients without alexithymia, those with alexithymia had more severe dysfunction with regard to immediate memory (F = 5.185; df = 1,693; p = 0.023), language (F = 9.767; df = 1,693; p = 0.002), and delayed memory (F = 4.844; df = 1,693; p = 0.028) and had lower total index scores (F = 8.700; df = 1,693; p = 0.003).

**Table 2:** Cognitive variables of schizophrenia patients with or without Alexithymia.

RBANS	Total patients	With Alexithymia	Without Alexithymia	*F*	*df*	*P*-value
	695	(n=218, 31.40%)	(n=477, 68.60%)			
Immediate Memory	56.72 (± 15.059)	54.80 (± 14.399)	57.59 (± 15.287)	5.185	1,693	**0.023**
Visuospatial/Constructional	80.05 (± 17.873)	78.08 (± 17.611)	80.95 (± 17.938)	3.867	1,693	0.050
Language	80.74 (± 13.448)	78.40 (± 12.433)	81.81 (± 13.767)	9.767	1,693	**0.002**
Attention	78.49 (± 15.281)	77.43 (± 14.641)	78.98 (± 15.556)	1.536	1,693	0.216
Delayed Memory	65.24 (± 19.237)	62.87 (± 18.988)	66.32 (± 19.272)	4.844	1,693	**0.028**
Total	65.83 (± 13.292)	63.65 (± 12.893)	66.83 (± 13.364)	8.700	1,693	**0.003**

Significant differences are highlighted in bold.

RBANS, Repeatable Battery for the Assessment of Neuropsychological Status.

### Influence of comorbid alexithymia on cognitive function in patients with SCZ

We used Pearson correlation to investigate the relationship between the total TAS-20 score and the RBANS index and total scores in patients with SCZ. The results revealed a significant negative correlation between the total TAS-20 score and the RBANS index and total scores in patients with SCZ. After Bonferroni correction, the correlations between the total TAS-20 score and all RBANS index scores, except for visuospatial/constructional index scores, or RBANS total scores remained significant (α = 0.05/21 = 0.0024). **Table 3** shows the relationship between TAS-20 scores and RBANS index and total scores. However, alexithymia showed weak but statistically significant correlations with RBANS scores (range: r=−0.133 to −0.192, p<0.01), suggesting modest associations.

**Table 3:** Association between Alexithymia and Cognitive variables.

	1	2	3	4	5	6	7
1. Alexithymia	-						
2. Immediate Memory	-0.157**	-					
3. Visuospatial/Constructional	-0.097*	0.459**	-				
4. Language	-0.141**	0.346**	0.349**	-			
5. Attention	-0.133**	0.413**	0.425**	0.349**	-		
6. Delayed Memory	-0.156**	0.660**	0.483**	0.344**	0.438**	-	
7. RBANS Total Score	-0.192**	0.789**	0.748**	0.588**	0.679**	0.821**	-

RBANS, Repeatable Battery for the Assessment of Neuropsychological Status.

*p < 0.05, **p <0.01.

Furthermore, stepwise multivariate regression analysis was performed for all patients to investigate the effects of alexithymia on cognitive function in patients with SCZ. The results showed that TAS-20 scores (β = -0.079, t = -2.191, 95% CI = [-0.210, -0.012], p = 0.029), educational qualification (β = 0.107, t = 3.010, 95% CI = [0.158, 0.749], p = 0.003), and PANSS general psychopathology scores (β = -0.122, t = -2.633, 95% CI = [-0.353, -0.051], p = 0.009) were associated with the RBANS language index score. In addition, TAS-20 scores (β = -0.077, t = -2.340, 95% CI = [-0.196, -0.017], p = 0.020), age (β = -0.073, t = -2.291, 95% CI = [-0.145, -0.011], p = 0.022), and BMI (β = 0.069, t = 2.139, 95% CI = [0.017, 0.394], p = 0.033) were associated with the RBANS total score.

## Discussion

This study revealed the effects of comorbid alexithymia on cognitive function in Han Chinese patients with chronic SCZ. The main findings of this study are as follows: (1) The prevalence of comorbid alexithymia in patients with SCZ was 31.40%. Symptoms of alexithymia were associated with some clinical features, including the PANSS negative symptom score. (2) Compared with patients without alexithymia, those with alexithymia had worse neurocognitive deficits with regard to immediate memory and delayed memory and had lower RBANS total scores. (3) Alexithymia was identified as a risk factor for language deficits and overall cognitive dysfunction (based on RBANS scores) in patients with SCZ.

The prevalence of alexithymia in patients with chronic SCZ observed in this study (31.4%) is consistent with that reported in previous studies from different cultures (5, 9, 26, 27). The prevalence of alexithymia in patients with chronic SCZ observed in this study (31.4%) is consistent with that reported in previous studies (5, 9, 14, 26–28) from different cultures. Notably, the prevalence of alexithymia observed in this study significantly exceeds that observed in healthy populations, which ranges from 5% to 25.3% (29–31). This finding suggests that patients with SCZ are at an increased risk of developing difficulties in identifying and expressing emotions. The increased prevalence of alexithymia may be partially attributed to the overlapping symptoms of alexithymia and schizophrenia, particularly concerning emotional blunting, emotional disengagement, and negative symptoms (32). In this study, patients with alexithymia had a higher number of hospitalizations, which may have contributed to their extended hospital stays. Prolonged social isolation and limited interpersonal interactions during these extended hospitalizations could have further exacerbated the challenges associated with emotional recognition and expression (33).

A notable finding of this study is sex disparity in the prevalence of alexithymia, with the proportion of male patients (35.34%) being higher than that of female patients (23.37%). This difference has been reported in many previous studies (9, 34, 35). The higher risk of alexithymia in male patients can be explained as follows: First, according to the Normative Male Alexithymia (NMA) hypothesis proposed by Levant, men raised within traditional paradigms are subject to emotional constriction, which impedes their ability to articulate emotions and feelings verbally. These traditional norms, ostensibly geared toward reinforcing power dynamics and concealing vulnerabilities within patriarchal social structures, may increase the risk of alexithymia in men (36). Second, empirical evidence indicates that compared with female patients with SCZ, male patients with SCZ exhibit more pronounced negative symptoms (e.g., anhedonia and avolition) and diminished social functioning (37–39), both of which are closely associated with dysphoria. However, contradictory findings have also been reported (26, 29), necessitating further investigation into the sex-specific risk of alexithymia to validate the findings of this study.

This study showed a significant correlation between alexithymia and the clinical symptoms of SCZ, particularly emphasizing the potential of alexithymia as an independent risk factor for negative symptoms (5, 9, 26). This relationship may be explained based on neurobiological alterations, specifically the changes in the structure and function of the amygdala, which are involved in the development of negative symptoms in SCZ (40). Changes in brain structure and function may also serve as the neurobiological basis of alexithymia (41). The salience network in the brain is responsible for the negative symptoms of SCZ, and reduced functioning of the salience network has been associated with alexithymia (42). These neurological impairments have led to the identification of a relationship between alexithymia and negative symptoms. Furthermore, personality traits may contribute to this relationship. Specifically, negative symptoms have been significantly positively correlated with the severity of depressive symptoms and the personality trait of harm avoidance (43). Previous studies have also shown that harm avoidance independently predicts alexithymia (43, 44). However, the findings of this study are not consistent with those of previous studies. Some studies have shown no correlation between alexithymia and negative symptoms (19, 32), whereas others have shown a correlation between alexithymia and positive symptoms (45, 46). Additionally, alexithymia has been positively correlated with the severity of hallucinations (19). In a longitudinal study, alexithymia in outpatients with SCZ was not found to be associated with negative symptoms. Moreover, the study showed significant improvements in the severity of negative symptoms but no significant changes in the severity of alexithymia over time (32). These discrepancies highlight the complexity of the relationship between alexithymia and SCZ, emphasizing the necessity of maintaining methodological uniformity in future studies.

Consistent with previous studies (9, 18, 21), this study showed that patients with SCZ with alexithymia had significantly poorer neurocognitive function. Alexithymia is a personality trait that affects the cognitive–emotional interaction (20). Therefore, it may be an important factor contributing to cognitive impairment in patients with SCZ. Furthermore, neurophysiological and neuroimaging studies have shown that cognitive dysfunction and alexithymia may stem from a common neural basis, specifically the corpus callosum, cingulate gyrus, orbital frontal cortex, and insulae. These regions are involved in the regulation of emotions (47, 48). However, some studies have shown a correlation between only a specific score and cognitive performance in alexithymia. For instance, a study showed that only the TAS-20 externally oriented thinking subscale score was associated with basic cognitive functions, such as working memory, attention, and spatial visualization abilities. The study found no relationship between difficulty in identifying feelings and most neurocognitive domains, except for attention (19). Fogley et al. used data from the Measurement and Treatment Research to Improve Cognition in Schizophrenia (MATRICS) project and found that deficits in processing speed, working memory, and abstract thinking were associated with DIF and EOT (18). However, no relationship was observed between cognitive deficits and DDF.

In this study, we found that patients with SCZ with alexithymia had worse cognitive function with regard to immediate and delayed memory. Alexithymia has been associated with memory. In a study, the memory of healthy individuals was assessed using the Rivermead Behavioral Memory Test (RBMT) Story Subtest. The results showed that individuals with a higher degree of EOT had poorer delayed memory (12). Nielson and Meltzer used an incidental memory task to investigate the impact of physiological arousal on memory in patients with alexithymia. Specifically, they compared the memory for neutral words between patients with high and low degrees of alexithymia. On an immediate recall task, patients with a high degree of alexithymia exhibited more severe memory impairments than those with a low degree of alexithymia (49). Another study showed that alexithymia led to impairments in immediate memory (50). DiStefano and Koven used the Wechsler Memory Scale-III, a non-emotional neuropsychological test, and found that patients with a low degree of alexithymia had better immediate and delayed visual memory than those with a high degree of alexithymia (51). These findings suggest an important relationship between alexithymia and both immediate and delayed memory. While univariate analyses suggested associations between alexithymia and memory deficits, these relationships were attenuated in multivariate models accounting for negative symptoms and other confounders. This indicates that the observed memory differences in univariate tests may reflect shared variance with clinical symptoms (e.g., PANSS negative scores) rather than a direct effect of alexithymia. These results highlight the importance of controlling for psychiatric symptoms when investigating cognitive correlates of alexithymia in schizophrenia. In this study, alexithymia was identified as a risk factor for language impairment. A study showed that patients with language impairment caused by a penetrating brain injury had more severe alexithymia than healthy individuals. In addition, the extent of brain damage in the inferior frontal gyrus (IFG), a key part of the language network, was associated with a higher degree of alexithymia (52). These findings are consistent with the linguistic hypothesis of alexithymia (53), which states that language impairments may contribute to the development of alexithymia. Therefore, further investigation is warranted to assess whether therapeutic interventions aimed at mitigating language impairments may alleviate alexithymia in patients with SCZ.

Despite notable findings, this study has some limitations that should be acknowledged. First, the use of a cross-sectional study design limited the in-depth investigation of the causal relationship between SCZ with comorbid alexithymia and cognitive impairment or negative symptoms. Long-term longitudinal studies may provide a more comprehensive understanding of this relationship. Second, this study included only Han Chinese patients with chronic SCZ. Therefore, further studies are warranted to confirm the relevance of the findings in patients with acute schizophrenia and diverse cultural backgrounds. Various medications and treatment factors can influence alexithymia and may obscure its relationship with clinical symptoms and cognitive functioning. Third, we did not thoroughly analyze the relationship between the individual TAS-20 subscales and cognitive functioning. Additionally, the severity of alexithymia was assessed solely based on self-reported questionnaires, without the use of clinical interviews. Fourth, although the RBANS tool enables a valid and reliable assessment of neurocognition, it does not include executive functioning, which is closely associated with alexithymia. Therefore, to validate and strengthen the findings of this study, comprehensive instruments should be used to assess affective disorders and neurocognition in future in-depth longitudinal studies. Fifth, we did not assess whether participants had sought or received psychotherapy or counseling interventions, which may have had an impact on their symptomatology. In future studies, we will aim to incorporate assessments of these non-prescriptive interventions to better understand their role in preventing or alleviating symptoms in individuals with schizophrenia.

In conclusion, this study revealed not only the prevalence of alexithymia in Han Chinese patients with chronic SCZ but also its significant relationship with negative symptoms and neurocognitive deficits. These findings suggest that early alexithymia screening should be incorporated into the diagnostic and therapeutic framework of schizophrenia and personalized treatment strategies should be developed to address challenges associated with comorbid alexithymia. In addition, future studies should focus on elucidating the pathological mechanisms underlying alexithymia in patients with SCZ and developing innovative treatment modalities for enhancing the well-being and quality of life of patients with schizophrenia with comorbid alexithymia.

## Data Availability

The original contributions presented in the study are included in the article/supplementary material. Further inquiries can be directed to the corresponding author.
